# Targeting the Dectin‑1 Receptor in Neuroinflammation: Therapeutic Implications for Neuropsychiatric Disorders

**DOI:** 10.1021/acschemneuro.5c00221

**Published:** 2025-08-04

**Authors:** Edna F. do Nascimento, Pauliane V. C. Batista, Carla S. Cunha, Luanny R. A. Lacerda, Vanessa C. Moura, Tatiana de Q. Oliveira, Adriano J. M. C. Filho, Pedro H. F. de Rezende, Danielle S. Macedo, Silvânia M. M. Vasconcelos

**Affiliations:** Drug Research and Development Center, 28121 Federal University of Ceará, Fortaleza 60430-275, Brazil

**Keywords:** CLEC7A, microglia, neuroinflammation, immunomodulation, neuropathology, central nervous system

## Abstract

Exploring the role of the pattern recognition receptor Dectin-1 in neurological diseases emerges as an important target for understanding the biochemical and physiological dynamics of neuropathologies. From this perspective, Dectin-1, protein encoded by the CLEC7A gene, stands out for its important role in antifungal immunity; however, the receptor also proves crucial in enabling the immune response of the central nervous system (CNS). This review highlights how Dectin-1 interacts with microglial cells, as well as the implications of these interactions in inflammatory, neurodegenerative, and psychiatric processes. In this regard, the narrative also revisits relevant discussions on the signaling pathways associated with Dectin-1, including the activation of tyrosine-protein kinase (Syk) and the production of inflammatory cytokines. It is noteworthy that altered expression of Dectin-1 has been observed in various conditions such as Alzheimer’s and Parkinson’s disease, thereby contributing to neuroinflammatory processes. However, in contrast to this, in depressive disorders, the receptor has shown the ability to modulate the inflammatory response, triggering antidepressant effects. Therefore, understanding the pluralistic role of Dectin-1 in the CNS may offer new scientific perspectives that will enable the development of more targeted therapies for neuroinflammatory and neurodegenerative diseases in different pathological contexts.

## Graphical abstract

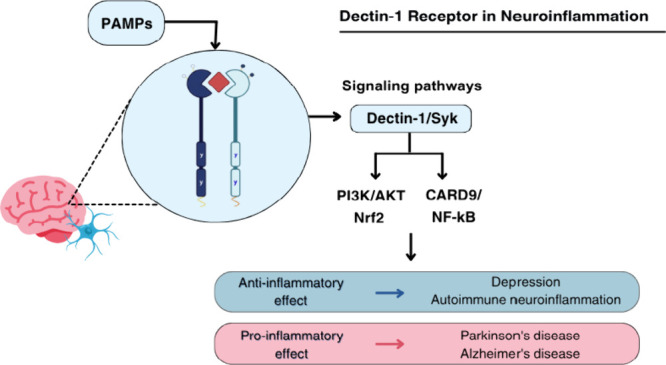

## 1 Introduction

The Dectin-1 receptor is a type II transmembrane lectin belonging to the C-type lectin receptor (CLR) family.^1^ This pattern recognition receptor (PRR) detects exogenous invading agents known as pathogen-associated molecular patterns (PAMPs), alerting the organism to infection presence.^2^ Moreover, it also recognizes intracellular molecules that are damaged or dying, referred to as damage-associated molecular patterns (DAMPs).^2^ The recognition of PAMPs and DAMPs influences the innate immune response by triggering different signaling pathways, phagocytosis, and the release of inflammatory mediators.^1^

Dectin-1 is widely known for its role in antifungal immunity;^1,3^ however, recent discoveries indicate that this receptor plays a broader role in defending against various pathogens, extending beyond fungi and bacteria.^1^ Furthermore, studies have shown that Dectin-1 is involved in sterile inflammation processes in the central nervous system (CNS).^2,4,5^ Thus, in addition to cells of the peripheral immune system, Dectin-1 is also expressed in CNS-resident cells, such as microglia.^6^ Consequently, the receptor has become the focus of recent research suggesting its role in regulating the neuroimmune system.^4^

The identification of Dectin-1 in CNS-resident cells highlights its role in neuroimmune regulation and, consequently, its potential involvement in neurodegenerative and neuroinflammatory pathogenesis, as well as its key role in disease-associated microglia (DAM). The function of Dectin-1 varies across different neurological diseases, depending on the pathological context. Studies show that Dectin-1 expression increases in response to brain injury and ischemia.^7,8^ Blocking the receptor minimizes macrophage-mediated axonal damage and reduces microglial activation.^9^ Conversely, in studies on major depressive disorder, Dectin-1 has been shown to modulate the brain’s anti-inflammatory response by reducing the production of pro-inflammatory cytokines IL-1β and TNF-α, which are associated with the development and progression of depression.^10^ In neurodegenerative diseases such as Parkinson’s and Alzheimer’s, evidence suggests that Dectin-1 contributes to neuroinflammation.^11–13^

The recent exploration of Dectin-1 in neurological diseases suggests a broad defensive role, especially when associated with microglial cells. This review aims to provide a comprehensive overview of the Dectin-1 receptor, from its pathological to molecular aspects, highlighting its main ligands and associated signaling pathways, as well as its implications in psychiatric disorders, autoimmune neuroinflammation, and neurodegenerative diseases. Furthermore, this study emphasizes the receptor’s role in neuropathologies to further elucidate its function in the neuroimmune system and propose potential new pharmacological targets for treating these disorders.

### 1.1 Structure and Distribution of the Dectin-1 Receptor

Dectin-1 possesses a complex molecular structure that functions as a sophisticated sensor, capable of initiating specific immune responses upon the detection of pathogenic agents (Figure 2). The receptor’s architecture comprises a C-terminal domain, a carbohydrate recognition domain (CRD), a short stalk region, a single transmembrane domain, and a short intracellular tail of about 40 amino acids.^14^

The Dectin-1 receptor, protein encoded by the CLEC7A gene, exists in eight documented isoforms, designated A through H (Figure 1).^15–17^ Isoform A is considered the main one, as it includes the full Dectin-1 structure, comprising the CRD, C’ to N’ terminal, stalk, transmembrane region, and immunoreceptor tyrosine-based inhibitory motif (hemITAM).^1,16^ Isoform B is structurally similar to A but lacks the stalk. However, both are considered complete isoforms and probably have the same ligand recognition mechanism, although their functional equivalence is not completely elucidated in biological systems, notably on the CNS. Isoforms C and D lack the CRD due to deletions. Isoforms E and F lack both the stalk and transmembrane region, while G and H contain some insertions in the CRD and transmembrane regions.^1^

The stalk region of the receptor plays an essential role in fungal detection and cellular response. Ligand recognition occurs through the extracellular CRD, a common feature among CLRs, which binds to sugars in a calcium-dependent or independent manner.^2^ Structurally, Dectin-1 possesses a single CRD that recognizes β-(1,3)/(1,6)-glucans, components of fungal and bacterial cell walls.^18^ However, the receptor lacks conserved residues necessary for calcium-dependent carbohydrate binding in the CRD, indicating that its ligand recognition is calcium-independent.^1^

The receptor also contains two critical cytoplasmic domains involved in signal transduction and interactions with intracellular proteins: the C-terminal cytoplasmic domain and the hemITAM and ITAM (immunoreceptor tyrosine-based activation motif) signaling motifs.^1^ The signaling motif follows the transmembrane region and is linked to the cytoplasmic tail.^1^ Genomic studies indicate that tyrosine is not essential for Dectin-1 signaling, which is characterized as hemITAM.^14,19^ Unlike ITAM, which contains two phosphorylated tyrosine residues to activate the tyrosine-protein kinase (Syk) signaling pathway, hemITAM has only one tyrosine, activating downstream signaling through a dimerization process in which the CRDs of the monomers form a PAMP-binding cavity (Figure 2). Additionally, researchers suggest that for ligand–receptor interaction, conserved amino acids (tryptophan 221 and histidine 223) are required to form the binding site and maintain receptor function.^2,20^

**Figure 1:**
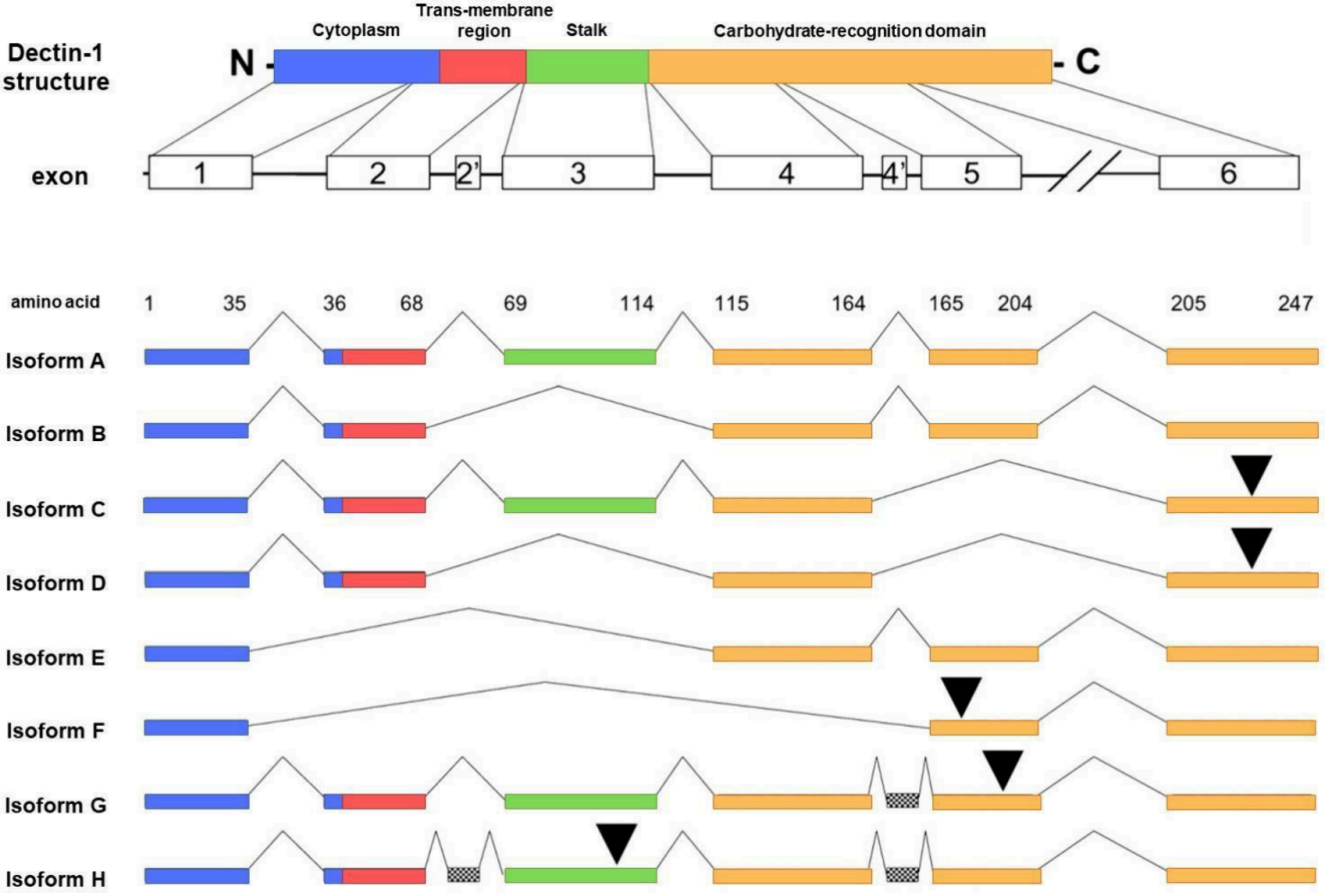
This scheme (adapted from Willment et al., 2001 and Kalia et al., 2021) illustrates the modular organization of the Dectin-1 protein and the structural variability among its eight isoforms (A–H) resulting from alternative splicing of the *CLEC7A* gene. Premature stop codons introduced by frameshift mutations are indicated by black arrowheads. Isoforms G and H incorporate additional exonic sequences within introns 2 and/or 4, which are shown with a checkered pattern.

### 1.2 Variations in Dectin-1 Receptor Expression

#### 1.2.1 Variation Among Cell Types

Dectin-1 is primarily expressed on the surface of nonspecific myeloid immune cells: macrophages, monocytes, neutrophils, and dendritic cells.^6,21^ Studies suggest that Dectin-1 expression in immune cells plays a role in immune tolerance, although the precise mechanisms have not been fully elucidated and more research is needed to draw more assertive conclusions.^22^ The receptor can also be found in lymphoid lineage cells such as gd T cells and epithelial cells like Langerhans cells.^23,24^ Evidence indicates that monocytes and monocyte-derived macrophages express Dectin-1 on their surface.^25^ Recent in vitro studies have also detected receptor expression in peripheral nervous system macrophages.^26^ Studies have shown that the expression of Dectin-1 in macrophages promotes an inflammatory environment.^27^ Although its expression is primarily in macrophages in this model, its activation significantly recruits neutrophils, supporting evidence that these cells functionally express the receptor.^28^

Regarding the CNS, immune system, Dectin-1 has also been identified in microglia.^6^ Studies demonstrate that primary mouse microglia can also express Dectin-1, particularly under inflammatory or neuropathological conditions.^6,12,29^ Under homeostatic conditions, however, microglia exhibit low levels of Dectin-1 expression, which can increase upon immune stimulation.^2^ Both in vivo and in vitro evidence support these findings, demonstrating microglial reactivity mediated by Dectin-1 signaling, as well as its presence in retinal microglia, where it modulates inflammatory pathways and phagocytosis in the context of antifungal defense responses.^22,25^ The receptor has also been identified at low levels in homeostatic microglia within the corpus callosum, cerebellum, white matter, and neurogenic niches.^30^ The broad expression of Dectin-1 in immune system cells highlights its functional versatility in the innate immune response.

#### 1.2.2 Interspecies Differences in Dectin-1 Expression

Dectin-1 expression can vary depending on the species. mouse and human Dectin-1 homologues share similar structures and functions.^31^ These homologues are involved in β-glucan pattern recognition and intracellular signaling through their cytoplasmic domain. However, they differ in expression patterns and regulatory mechanisms.^32^ In mouse Dectin-1, N-linked glycosylation occurs in the C-type lectin domain and only in full-length receptor β-glucan stalks. Nonetheless, evidence indicates that these receptors share 60% sequence identity and 71% sequence similarity, therefore, suggesting a conserved evolutionary origin and similar functions, respectively.^33^ Thus, mouse and human Dectin-1 exhibit similar functional characteristics and may downregulate the receptor’s costimulatory function.^32^ Still, there are differences in the expression levels and regulation of Dectin-1 in mouse and human dendritic cells. In humans, Dectin-1 expression appears to be modulated in a context-dependent manner. Studies show that during in vitro exposure to the pathogenic fungus Aspergillus fumigatus, certain conditions of innate immune cells show a reduction in Dectin-1 expression, suggesting a possible regulatory mechanism distinct from those observed in mice.^34^ Furthermore, unlike mice, humans express eight Dectin-1 isoforms, resulting in two major isoforms (A and B) and six minor ones. In mice, Dectin-1 is predominantly expressed by myeloid and dendritic cells.^31^ The human homologue, however, is also found in B cells, eosinophils, neutrophils and mast cells.^31^ Humans deficient in Dectin-1 are more susceptible to fungal infections.^3^ The DECTIN1 Y238X variant (rs16910526) reduces surface expression of Dectin-1 and is associated with vulvovaginal candidiasis and increased risk of fungal infections in immunocompromised patients.^35,36^ Furthermore, studies have identified the presence of an O-glycosylation-rich tail in humans, absent in mice, which serves as a ligand for the CLEC2 gene, another CLR.^37^ In other animals, such as horses and cattle, functional isoforms of Dectin-1 have been identified that are capable of stimulating inflammatory signaling in response to fungal infection.^38,39^

#### 1.2.3 Influence of Age on Dectin-1 Expression

The immune response induced by Dectin-1 is also age-related. A comparative study of innate immunity between young and aged mice showed that Dectin-1 expression affects innate immune responses during aging.^40^ Other evidence has reported similar results when comparing Dectin-1 expression in primary human monocytes and monocyte-derived dendritic cells (moDCs) from young and adult individuals with human immunodeficiency virus (HIV), revealing differences in receptor responses.^41^ Findings show that older individuals exhibit a more robust inflammatory response following Dectin-1 stimulation, with increased production of inflammatory cytokines suggesting an enhanced immune response in advanced age.^41^ These have also been identified in studies of inflammatory stimulation of Dectin-1, in which inflammation caused by the receptor appears to contribute to the pro-inflammatory environment in both aging and HIV infection.^42^ However, there are still gaps in the literature that require further investigation to elucidate how Dectin-1 expression is regulated throughout developmental stages.

## 2 Dectin-1 as the Main Receptor for β-Glucans

This PRR, Dectin-1 is the primary receptor for β-glucans on the surface of host cells.^20^ The β-(1–3)/(1–6) linkages present in glucans are recognized by the receptor and are characteristic of polysaccharides found in the cell walls of pathogens, including fungi and bacteria.^20^ In addition to the β-(1–3)/(1–6) linkages, β-glucans exhibit heterologous structural differences that determine their association with the receptor.^2^ Factors such as ligand molecular size, polymer length, solubility, and branching are essential for triggering immune responses mediated by Dectin-1.^2^ Components of the glucan group that binds to Dectin-1 include zymosan, Curdlan, whole glucan particles (WGP), lentinan, and laminarin the latter two being soluble.^43^

Zymosan can modulate Dectin-1 signaling, inducing a strong inflammatory response.^44^ It acts as a Dectin-1 receptor agonist, promoting the release of IL-2, IL-10, IL-12p70, TNF-α, IL-6, reactive oxygen species (ROS), and triggering phagocytosis.^44,45^ However, it can also act as an anti-inflammatory agent by suppressing autoimmune neuroinflammation.^46^ Other glucans are more effective in signaling inflammatory responses. For example, studies show that Curdlan induces greater IL-1β transcription and secretion compared to zymosan and paramylon.^43^ Curdlan functions as a Dectin-1 agonist by activating pro-inflammatory signaling pathways, evidenced by increased production of ROS, IL-2, TNF-α, and costimulatory molecules.^47–49^ Curdlan β-glucan acts on mast cells and dendritic cells and is a major target in studies investigating enhanced antitumor immunity through dendritic cell activity.^47–49^

Particle size is another determining factor in Dectin-1 signaling. According to Elder et al., 2017, larger β-glucan molecules stimulate higher production of IL-1β, IL-6, and IL-23 in dendritic cells compared to smaller molecules.^50^ Furthermore, Dectin-1 signaling is activated by particulate ligands, not by soluble glucans.^34^ Soluble β-glucans do not induce the formation of a phagocytic synapse.^45^ However, studies involving laminarin linear, soluble β-glucan have observed anti-inflammatory potential through modulation of the Dectin-1 receptor.^19,44,41^ Laminarin reduces levels of pro-inflammatory cytokines and can act as a blocking agent against other β-glucans, such as zymosan and alternative substrates. It also shows high-affinity binding to the receptor.^2,19^ Recent evidence highlights the anti-inflammatory potential of laminarin in reducing microglial cell activation by downregulating receptor signaling, functioning as a Dectin-1 antagonist.^51^ Other studies have also identified the action of laminarin as a Dectin-1 antagonist, suppressing the development of colored tumors in mice.^52^ However, in research involving , laminarin acts as a receptor agonist and inhibits chlamydia infection in cervical epithelial cells and in mice.^53^

Other soluble glucans, such as lentinan, have also shown anti-inflammatory activity by inducing robust IL-10 production and brain-derived neurotrophic factor (BDNF). However, unlike laminarin, lentinan upregulates the expression of the receptor.^54^ These findings suggest that ligand molecular size, length, branching, and solubility are key factors that condition receptor modulation during immune responses.^2^ Nonetheless, beyond β-glucans, other ligands are currently being documented for their ligand–receptor interactions and influence on the immune system. Dectin-1-associated DAMPs, such as annexin, vimentin, and galectin-9, are being studied as therapeutic targets for diseases such as cancer, atherosclerosis, autoimmune disorders, and in contexts like aging and obesity.^55–57^

## 3 Signaling Pathways

The activation of Dectin-1 triggers an inflammatory response through cytokine expression and stimulation of phagocytosis. These inflammatory signals, induced by the presence of PAMPs and DAMPs, can be detected by Dectin-1 via its hemITAM component.^2^ When a ligand is detected, the receptor stimulates the protein tyrosine kinase family (SRC) to phosphorylate ITAM on tyrosine residues, enabling Syk binding and initiating intracellular signaling (Figure 3).^2,58^ Syk is a central tyrosine kinase, predominantly expressed in microglia, and is associated with various inflammatory pathways.^59^ While Syk mediates most of Dectin-1’s functions, there are also cell-type-specific signaling pathways independent of the direct receptor-protein interaction.^1^ Upon ligand detection in the cellular environment, a conformational change occurs in the receptor, triggering the clustering of hemITAMs in the cytoplasmic tail.^2^ This interaction with Syk activates enzymatic activity and initiates signal transduction.^2^ Several signaling pathways can be triggered depending on Dectin-1’s Syk-dependent interaction.^1^

**Figure 2:**
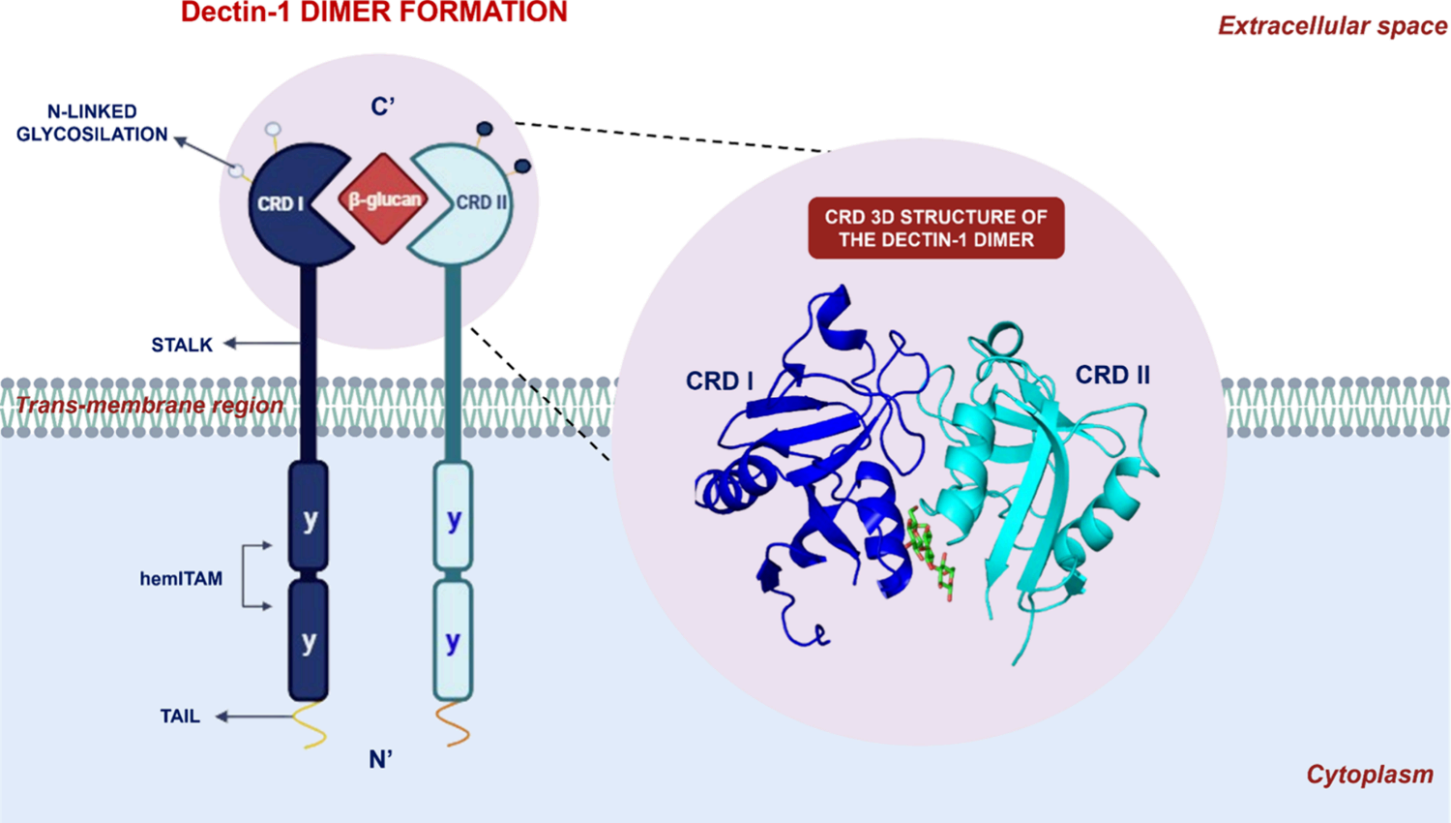
Graphical illustration of the complete structure of a Dectin-1 dimer. The Dectin-1 receptor is characterized by an intracellular cytoplasmic domain containing a hemi-ITAM, a transmembrane region, and an CRD. The CRD is the specific portion of the receptor responsible for binding to β-glucan ligands. While Dectin-1 can exist as a monomer in the absence of a ligand, upon ligand binding, it forms a dimer. In this dimeric state, the CRD of monomer I (CRD I) and the CRD of monomer II (CRD II) cooperatively create a high-affinity interaction cavity for the carbohydrate. This dimerization is crucial as it facilitates the recruitment and subsequent activation of the Syk to the ITAM within the cytoplasmic domain. This initiation of Syk signaling then triggers a downstream intracellular signaling cascade, orchestrating the Dectin-1-mediated immune response. Structure from PDB code RCSB – 2 CL8.

### 3.1 PI3K/AKT

Part of the inflammatory response generated via the Syk/Dectin-1 axis involves the PI3K/AKT pathway.^60^ Phosphatidylinositol 3-kinases (PI3Ks) and their downstream effector protein kinase B (AKT) are key components in intracellular signaling in response to extracellular stimuli.^61^ Upon ligand recognition, Dectin-1 mediates PI3K/AKT activation through Syk.^62^ This pathway is activated in response to β-glucan stimulation.^62^ When the β-glucan molecule is larger, monocytes enhance cytokine and ROS production.^50,63^ Independently of Syk, Dectin-1 can also activate the serine-threonine kinase Raf-1, which converges with the Syk pathway at the NF-κB (nuclear factor kappa B) level, both being involved in phagocytosis through PI3K/AKT.^2^ Raf-1 signaling promotes trained immunity induced by β-glucan detection. Dectin-1-mediated trained immunity is currently being explored as a therapeutic strategy.^2,64^ The PI3K/AKT and Raf-1 pathways are essential for cytokine production following DAMP recognition and Zymosan phagocytosis.^65^ It is important to note that Dectin-1-mediated Syk activation also results in the activation of Nuclear Factor of Activated T Cells (NFAT) in dendritic cells and macrophages.^4,44,66^ With the activation of the Syk pathway, there is an increase in intracellular calcium (Ca^2+^) levels through PLC-γ2, promoting the activation of NFAT and, subsequently, also activating the expression of the early growth response (Erg) transcription family, cyclooxygenase-2, IL-2 and IL-10.^4,44^

### 3.2 CARD9/NF-kB

Downstream signaling from Syk also involves the caspase recruitment domain-containing protein 9 (CARD9). CARD9 is an adaptor protein found in myeloid cells, crucial in fungal infections.^67^ CARD9 forms an activating complex for nuclear factor kappa B (NF-κB) through interactions with B-cell lymphoma/leukemia 10 (BCL10) and mucosa-associated lymphoid tissue 1 (MALT1).^68,69^ This trimolecular complex leads to the production of inflammatory cytokines such as pro-IL-1β, IL-6, and TNF-α.^1,68^ The CARD9/BCL10/MALT1 complex mediated by the activation of the Syk pathway contributes to the activation of mitogen-activated protein kinases (MAPK) and consequently the production of cytokines.^70^ Dectin-1 also initiates another Syk-dependent signaling pathway that results in the production of type I interferons (IFN-I).^1^ IFN-I represents a family of soluble mediators activated by Dectin-1 upon detecting Candida and Dectin-1 receptor agonists like Curdlan.^71^ Additionally, signaling molecules involved in IFN-I production include not only Dectin-1 and Syk but also CARD9 and IRF-5 (interferon regulatory factor 5).^71^ Evidence indicates that IRF5 activation was triggered by Dectin-1 after recognition of glucans in tumor cells.^72^ In general, IRFs are characterized as transcription factors allocated in the cytosol that, when migrating to the nucleus, contribute to the transcription of target genes.^2^ IRF5 is constitutively expressed in certain immune cells and becomes activated in response to signals from endosomal Toll-like receptors (TLRs), especially TLR7, TLR8, and TLR9.^73^ Studies indicate that the production of IFR5 with Toll-like receptors (TLRs) in endosomes requires the phosphorylation of IRF-5 by kinases such as Transforming Growth Factor b-Activated Kinase 1 (TAK1) and Nuclear Factor Kappa-B Inhibitory Kinase Beta (IKKβ).^73^ Once phosphorylated, IRF5 translocates to the nucleus and induces the expression of pro-inflammatory cytokines and type I interferons.^73^

### 3.3 Nrf2

Dectin-1 plays a key role in ROS generation, which is essential for antifungal defense in macrophages. However, Dectin-1 also acts through the nuclear factor erythroid 2–related factor 2 (Nrf2) and intracellular heme oxygenase 1 (HO-1) pathway.^74,75^ Nrf2 is a nuclear transcription factor that regulates genes encoding antioxidant enzymes and proteins.^74^ The PI3K/AKT pathway promotes Nrf2 translocation to the nucleus and regulates antioxidant enzyme signaling.^76,77^ In immortalized murine macrophages, such as RAW 264.7 cells, the β-glucan receptor reduces ROS production stimulated with LPS.^78^ In addition to reducing ROS levels, Nrf2/HO1 increased the production of antioxidant enzymes, such as superoxide dismutase (SOD), catalase (CAT), and glutathione peroxidase (GSH-Px).^78^ In contrast, evidence has identified the anti-inflammatory role of Nrf2/HO-1 through the inhibition of Dectin-1, reducing corneal inflammation with the activation of this pathway.^79^ Other studies involving inflammatory bowel diseases suggest that Dectin-1 activation contributes to oxidative stress, which may influence the activation of antioxidant pathways, such as Nrf2/HO1.^80^ Thus, although Dectin-1 acts in the inflammatory response, it also demonstrates antioxidant properties, being a promising target to alleviate oxidative stress and provide new natural compounds for the treatment of ROS-related diseases.^78^ However, the precise involvement of Dectin-1 in the modulation of the Nrf2/HO1 pathway is not yet completely established, requiring further studies to be elucidated.

## 4 Dectin-1 as a Marker in Central Nervous System Disorders

Dectin-1 is well documented for its role in antifungal immunity, including responses against Aspergillus, Candida, Coccidioides, Pneumocystis, and Saccharomyces. This PRR has also been implicated in respiratory diseases and cancer. However, its role in the CNS remains underexplored, leaving gaps in the understanding of its therapeutic potential. This section explores the involvement of the Dectin-1 receptor in various neurological disorders, including psychiatric conditions such as depression, autoimmune neuroinflammation, and neurodegenerative diseases such as Parkinson’s and Alzheimer’s (Table 1).

**Figure 3:**
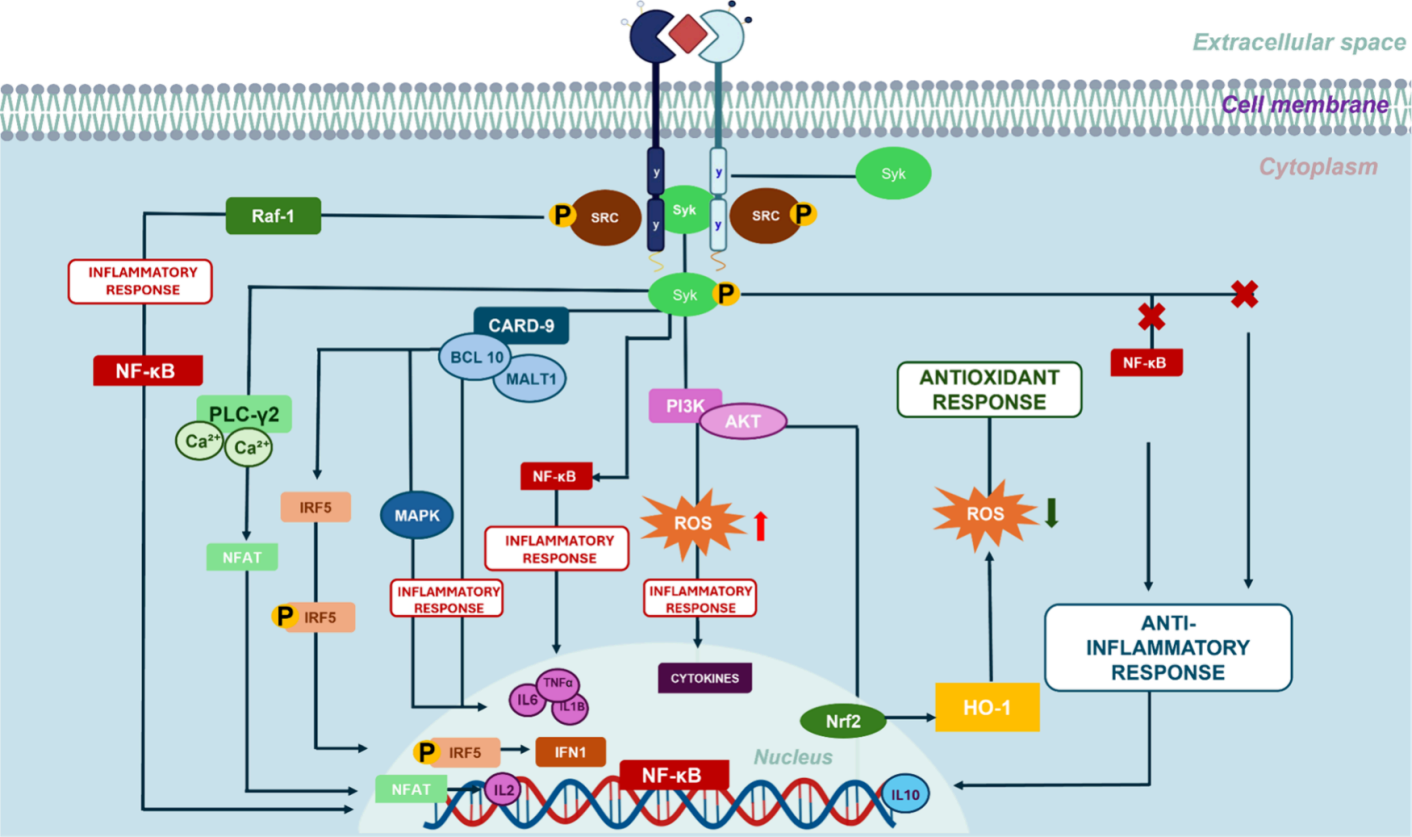
Dectin-1-mediated intracellular signaling after ligand recognition. Src kinases phosphorylate tyrosine residues of Dectin-1 and form a site for Syk, which is recruited to initiate the intracellular signaling. Syk/Dectin-1 promotes downstream activation through molecules such as CARD9, MALT1, and BCL10 for NF-κB activation and inflammatory cytokine production. Dectin-1 mediates NFAT and NF-κB activation independently of the CARD9 pathway. Dectin-1 also induces Syk-independent signaling mediated by Raf-1. Blockade of the Syk and Syk/NF-κB pathways enables the production of anti-inflammatory cytokines such as IL-10.

**Table 1:** Dectin-1 Immunomodulation in Different CNS^a^

CNS diseases	neuropathology	study model	cell type of expression	Dectin-1 role	ref
neurodegenerative diseases	Alzheimer’s disease	in vitro	microglia	increased Syk signaling was observed through microglial activation by Clec7a in Aβ pathology. Blockade of Clec7a in microglia by the Anti-Clec7a antibody recovered part of the microglial response to Aβ pathology and Syk signaling. This response promoted increased phagocytosis of Aβ plaques, the number of dynamic and inert plaques, and reduced the number of filamentous plaques	^115^
				Aβ42 increased Dectin-1-Syk interaction in BV2 cells. In HEK-293 cells exposed to Aβ42, Dectin-1 homodimerized. Also, Aβ42 did not activate NF-κB or Syk phosphorylation in Dectin-1-deficient BV2 cells. Dectin-1 knockdown inhibited Aβ42-induced nuclear p65 translocation and reduced TNF-α and IL-1β levels and TNF-α, IL-1β, IL-6, Cox2 and iNOS expression. Dectin-1 also bound directly to Aβ42. In PC12 cells, microglia medium with Dectin-1 induces apoptosis and ROS production, effect reversed in the absence of the receptor	^12^
	Parkinson’s disease	i n vitro and in vivo	microglia	Clec7a was highly expressed in the substantia nigra (SN), striatum and reactive microglia of rats in a PD model. Knockdown of Clec7a improved motor symptoms, protected dopaminergic neurons and reduced levels of TNF-α, IL-1β, IL-6, IL-18 and IFN-γ in the SN and decreased microglial pro-inflammatory polarization. In a cell model of PD, Clec7a overexpression increased iNOS+ cells, while gene knockdown reversed this effect and reduced SN levels of TNF-α, IL-1β, IL-18 and IL-6	^112^
				Clec7a+ disease-associated microglia cell bodies of Lrrk2 G2019S mice presented structures related to oxidative stress, such as dilation of the Golgi complex. These microglia exhibited intense phagocytic activity, and were also seen with ingested protein aggregates. Clec7+ microglia were more abundant in the striatum of older mice. In aged Lrrk2 G2019S mice, microglia cells were clustering and presenting ameboid morphology. Also, Lrrk2 G2019S mice showed a higher proportion of Clec7a+ microglial cells compared to WT mice	^115^
neuroinflammation	Ischemic stroke	i n vitro and in vivo	BV2 microglia	Dectin-1 was highly expressed in ischemic brain tissue and BV2 microglia in the oxygen-glucose deprivation/reoxygenation model. Laminarin treatment reduced brain infarct volume and neurological deficits, suggesting Dectin-1’s role in stroke pathophysiology. Inhibition of Dectin-1 blocked Dectin-1/Syk signaling both in vitro and in vivo. Laminarin decreased TNF-α and iNOS levels in mice and BV2 cells. Also, this treatment decreased microglial activation in ischemic mice. Piceatannol (PIC), a Syk inhibitor, reduced brain infarct volume and neurological impairment in mice. Dectin-1 knockdown in BV2 cells lowered p-Syk, iNOS, and TNF-α levels, alleviating inflammation. In LPS-induced inflammation, inhibition of Dectin-1 or p-Syk by Laminarin or PIC reduced iNOS and TNF-α in BV2 cells	^81^
	Multiple sclerosis	in vitro	microglia BV2	Dectin-1 was expressed at higher levels in BV2 microglia after treatment with lentinan (LNT) than LPS. In LNT-treated cells, it is suggested that inhibition of Dectin-1 by the antagonist laminarin blocks the downregulation of Iba1, iNOS, TNF-α, and IL-1β, and the upregulation of Arg-1, IL-10, and BDNF, indicating that LNT may have anti-inflammatory action by Dectin-1	^54^
		in vivo	myeloid cells	Dectin-1 attenuated EAE severity in mice, while Clec7a–/– mice suffered immune cell infiltration into the spinal cord. Dectin-1 was more expressed in CNS-infiltrated myeloid cells than in microglia from EAE mice. Furthermore, myeloid cells appear to regulate Dectin-1 function during EAE. Curdlan-induced Oscostatin M (Osm) upregulation in myeloid cells showed how Dectin-1 mediates neuroprotective factors production, which occurs through a Card9-independent pathway that encompasses Syk, PLC, Ca^2^?, calcineurin and NFAT. Also, Osm receptor signaling in astrocytes and Gal-9 in myeloid cells limited EAE severity likely through Dectin-1	^84^
	Systemic lupus erythematosus (SLE)	in vivo	microglia and astrocytes	in two mouse models of lupus, FcγRIIB–/–Yaa and NZB/NZW strains, microglial cells exhibited upregulated expression of the Clec7a gene	^87^
mood disorder	Depression	in vivo	macrophage	Dectin-1 mRNA levels in the jejunal mucosa of animals increased after a high molecular weight β-glucan diet. It was observed that β-glucan regulates immune activity by clustering Dectin-1 receptors and inhibiting TLR4, reducing inflammatory cytokines (IL-1β, IL-6 and TNF-α) and increasing IL-10 levels	^83^
		in vivo	microglia	increased levels of Dectin-1 were found in the hippocampus of animals treated with the antidepressant substance GLP. In animals subjected to the chronic social defeat stress model, Dectin-1 levels were reduced in the hippocampus, an effect reversed after GLP administration. Laminarin, a Dectin-1 inhibitor, almost completely blocked the reduction in immobility time induced by GLP in the forced swimming test	^10^
	psychosocial stress	in vivo	γδ T cells	Dectin-1 was expressed in colonic γδ T cells, which were more frequent in mice vulnerable to stress. Clec7a–/– mice did not show an increase in γδ, γδ17, or CD8+ T cells and did not develop social-avoidance after chronic social-defeat stress (CSDS). Furthermore, TCRd-KO mice receiving γδ T cells lacking Dectin-1 showed a lower frequency of γδ and γδ17 T cells in the colon after stress. Only TCRd-KO mice receiving γδ T cells with Dectin-1 exhibited social-avoidance behavior after stress. Treatment with pachyman, a Dectin-1 ligand, inhibited the increase in the frequency of γδ, γδ17 and CD8+ T cells in the colon and reversed CSDS-induced social-avoidance, suggesting a role for Dectin-1 in mediating γδ T cell growth and γδ17 differentiation, as well as in behavioral changes	^108^

a Aβ42: amyloid beta peptide 1–42, Clec7a: gene that encodes Dectin-1 receptor, Clec7a–/– mice: mice with genetic deletion of Clec7a gene, CSDS: chronic social-defeat stress test, EAE: experimental autoimmune encephalomyelitis, GLP: polysaccharide, LNT: Lentinan, Osm: Oncostatin M cytokine, p65: subunit of the nuclear factor kappa B, PD: Parkinson’s disease, PIC: Piceatannol, ROS: reactive oxygen species, SN: substantia nigra, WT: wild-type animal.

### 4.1 Neuroinflammation

The Dectin-1 receptor plays a key role in neuroinflammation. In vitro studies suggest that activation of Dectin-1 receptors can promote remyelination through treatment with Lentinan (LNT), impacting axonal degeneration by inhibiting neuroinflammation and promoting the transformation of microglial cells from the M1 to the M2 phenotype.^54^ LNT improved anti-inflammatory markers such as IL-10 and BDNF, while inhibiting pro-inflammatory markers TNF-α and IL-1β by downregulating microglial activation and the proliferation of oligodendrocytes and astrocytes via Dectin-1 modulation.^54^ The regulatory inhibitory action of this receptor may reduce inflammatory responses.^54^ In ischemic stroke, Dectin-1 expression in microglia leads to increased Syk phosphorylation and expression of inducible nitric oxide synthase (iNOS) and TNF-α. This Dectin-1/Syk interaction plays a key role in inflammatory signaling.^81^ Blocking this receptor and its downstream effector Syk reduces inflammatory activity and decreases cerebral infarct volume.^81^ A recent study presented both in vitro and in vivo data demonstrating the role of CLEC7A in ischemic stroke, the pyroptosis cell process and microglia activation.^82^ Li et al. (2024) reported that knockdown of CLEC7A in BV2 microglia cells increased the viability of HT22 neuronal cells in a coculture system of HT22 and BV2 cells subjected to oxygen-glucose deprivation/reperfusion (OGD/R). In this coculture system with CLEC7A silencing, HT22 neuronal cells showed lower levels of IL-1β, IL-18, TNF-α, lactate dehydrogenase (LDH), and pyroptosis-related proteins gasdermin D (GSDMD), Caspase-1, and nod-like receptor protein 3 (NLRP3), compared to the group with CLEC7A silencing plus pyroptosis activator under OGD/R conditions. CLEC7A silencing also reduced infarct size and brain water content in ischemic stroke rats. Furthermore, CLEC7A knockdown in rats decreased the levels of IL-1β, IL-18, TNF-α, and LDH, as well as microglia activation and the levels of pyroptosis-related proteins.^82^

Conversely, in the animal model of experimental autoimmune encephalomyelitis (EAE), upregulation of Dectin-1 demonstrates anti-inflammatory potential.^82^ These results align with recent studies identifying the role of Dectin-1 in limiting EAE.^83^ Furthermore, Dectin-1 promotes beneficial crosstalk between myeloid cells and astrocytes via oncostatin M, and its pro-inflammatory response is mediated through CARD9/NF-κB.^61^ However, according to Deerhake et al. (2021), Dectin-1 also induces the expression of a neuroprotective cytokine and a transcriptional program with protective and anti-inflammatory functions, independent of CARD9.^84^ Nevertheless, this anti-inflammatory effect is not observed in mild experimental autoimmune uveitis (EAU).^85^ On the other hand, Zhang et al. (2023) suggested that microglial activation is associated with pathogenic Eomes? Th cells during the late phase of EAE. Inhibition of microglia abolished EAE symptoms, reduced levels of Eomes? Th cells in the CNS, and decreased IFN-1 gene expression in microglia. Moreover, the Clec7a gene was upregulated, and stimulation with its agonist zymosan induced IFN-1 production during late EAE.^86^ Evidence suggests that Dectin-1 contributes to pro-inflammatory responses, increasing IL-23 production, a process linked to pathogenic Th17 cell responses.^85^ Additionally, the gene encoding Dectin-1, CLEC7A, has been found in studies on Systemic Lupus Erythematosus (SLE).^87^ In lupus-prone mice, microglia upregulate genes associated with neurodegeneration and interferon responses, including CLEC7A.^87^ However, further studies are needed to fully understand the role of this receptor in neuroimmune responses.

However, despite the advances observed in the studies cited, there is a clear lack and need for clinical studies in neuroinflammatory conditions. The response in animals may exhibit substantial differences compared to human neuroinflammation. EAE shares immunological characteristics similar to those in humans, but differs in terms of cellular and pathophysiological response. Just like ischemia and lupus, which do not encompass the genetic and clinical complexity of the disease in humans. Thus, although preclinical studies provide indispensable findings for understanding the mechanisms of neuroinflammatory diseases, the translation of studies to the clinical scenario requires more robust preclinical models and humanized models.^88–91^

### 4.2 Disease-Associated Microglia (DAM)

Microglia are resident macrophages of the brain and constitute the first line of immune defense in the CNS.^92^ These cells play roles during development and adulthood, acquiring different phenotypes in response to environmental signals.^92,93^ Microglia are dynamic and capable of morphological changes in a short time frame.^94^ Due to this functionality, microglia are key players in brain injury and disease.^94^ In this context, DAM has become a major research focus for understanding the pathophysiology of neurological disorders. Cumulative evidence suggests a link between DAM and Dectin-1 in CNS diseases. Studies have identified CLEC7A expression in microglial subpopulations, making it one of the most highly upregulated genes in DAM.^95^

Neurodegenerative diseases such as Alzheimer’s disease (AD) have been linked to disease-associated microglia (DAM), and recent research has shown that the accumulation of human tau in animal model of AD can increase DAM levels.^96^ In AD, the TREM2 receptor (Triggering Receptor Expressed on Myeloid Cells 2) is a well-known surface receptor associated with DAM.^95^ Microglial expression of Dectin-1/Clec7a is a key feature of the TREM2-dependent stage.^97^ Initially, microglia are activated, downregulating genes involved in homeostasis and upregulating TREM2 and apolipoprotein E (APOE), which in turn upregulate CLEC7A and its signaling pathway.^97^ Notably, CLEC7A has been shown to compensate for TREM2 deficiency by addressing metabolic derailment and autophagy dysfunction.^98^ CLEC7A triggers intracellular signaling similar to TREM2 via Syk and PI3K activation, leading to autophagy suppression.^98^

A model for AD has been proposed by Dios et al. (2023) based on immune system modulation by cholesterol. In this model, inflammasome signaling in microglia and neurons is regulated by cholesterol levels, and microglia tend to shift toward a DAM phenotype in response to neuronal death driven by inflammatory conditions. This model is based in the author’s research results, in which it was observed that in the SIM-A9 microglial cell line, high cholesterol levels increase NLRP3 expression during inflammasome activation, upregulate TREM2 mRNA, and likely influence microglia to shift toward a DAM phenotype. Furthermore, exposure to LPS plus muramyl pipeptide (MDP), two inflammatory agents, associated with elevated cholesterol content, enhanced Clec7a mRNA levels in cultured microglia cells more than when these conditions were applied separately.^99^

In multiple sclerosis, Dectin-1 modulation inhibits neuroinflammation by converting microglia from the M1 to M2 phenotype, enhancing anti-inflammatory markers IL-10 and BDNF and reducing pro-inflammatory markers TNF-α and IL-1β.^54^ In ischemic stroke, Dectin-1 expression in microglia increases Syk phosphorylation and expression of iNOS and TNF-α, underscoring the key inflammatory signaling role of Dectin-1/Syk.^81^ Blocking this receptor and Syk results in reduced inflammation and decreased infarct volume.^81^

Additionally, the receptor is linked to retinal microglial diseases. In vivo evidence shows that Dectin-1 signaling in microglia can modulate ongoing neuroinflammatory responses to become more protective and pro-regenerative following axonal injury.^100^ These findings are consistent with in vitro spinal cord injury studies, where microglial reactivity is mediated by Dectin-1 signaling, increasing its pro-inflammatory activity in the absence of axons and myelin.^101^ This results in axonal injury and demyelination.^102^ Conversely, optic nerve crush models show that upregulation of Dectin-1 promotes enhanced axonal regeneration in retinal microglia and dendritic cells.^103^

Therefore, advancing knowledge of Dectin-1 and its roles in microglia supports a better understanding of neuroimmunology and the treatment of neuroinflammatory diseases.

### 4.3 Depression

Major depressive disorder is a persistent and multifaceted condition with complex pathophysiology. The development of novel antidepressant strategies is imperative, as currently available pharmacological treatments fail to achieve adequate therapeutic response in a significant proportion of patients. Cumulative evidence suggests that the Dectin-1 receptor plays an important role in the treatment of this mood disorder. Li et al. (2021) identified the rapid and robust antidepressant potential of Dectin-1 through activation by polysaccharide (GLP).^10^ Treatment with GLP attenuated the expression of potential markers of depressive disorder such as IL-1β and TNF-α and increased the expression of the anti-inflammatory cytokine IL-10 and brain-derived neurotrophic factor (BDNF) in the hippocampus of mice.^10^ Receptor levels significantly increased following GLP treatment, and Dectin-1 receptor blockers such as laminarin inhibited GLP’s antidepressant effect.^10^

Previous studies have identified the antidepressant potential of Dectin-1 through its activation by β-1,3-linked glucan.^104,105^ The observed antidepressant effect was robust and long-lasting due to increased receptor levels and the activation of the Dectin-1/AMPA signaling pathway.^105^ Binding to Dectin-1 can lead to activation of the Syk/NFκB signaling pathway and immune system regulation.^105^ Similar results also identified this pathway in the treatment of depression. The Dectin-1/AMPA receptor signaling pathway is linked to the immune system.^104^ Dectin-1 upregulates the expression of cytokines IL-2, IL-4, and IL-13, which promote synaptic plasticity, including the synaptic expression of AMPA a central mediator in the treatment of depression.^104^ However, a study investigating the anti-inflammatory effects of β-glucans found that the antidepressant effect of Dectin-1 may occur through the blockade of other associated receptors.^83^ Dectin-1 can inhibit the activation of the TLR4 receptor, resulting in decreased pro-inflammatory cytokines such as IL-1β, IL-6, and TNF-α, and increased production of anti-inflammatory cytokines like IL-10.^83^ According to the authors, higher molecular weight β-glucans are stronger activators of the Dectin-1 receptor, enhancing its response in the immune regulatory pathway, which in turn depends on TLR4 receptors.^83^ Additional in vivo studies, such as Zhao et al. (2024) and Ren et al. (2024), also provide relevant data that allow theoretical inference about the influence of the Dectin-1 receptor on depression, although the authors did not directly evaluate this relationship.^106,107^ In Zhao et al. (2024), stimulation of microglia by β-glucan administration in depressive mice reversed depressive-like behavior. Since β-glucan is a polysaccharide that interacts with the Dectin-1 receptor on microglia, it is assumed that the antidepressant effect is related to the activation of this receptor, notably on these cells.^106^ The study by Ren et al. (2024) also supports this assumption, as β-glucan, by stimulating the immune system, was able to prevent the emergence of depressive behavior and the increase of pro-inflammatory cytokines IL-1β, TNF-α, and IL-6 in the hippocampus and prefrontal cortex of mice in a chronic unpredictable stress animal model.^107^

In contrast, despite its anti-inflammatory effect in depression, recent studies have linked Dectin-1 to the development of psychosocial stress.^108^ According to Zhu et al. (2023), Dectin-1 receptor signaling modulates behavioral vulnerability to chronic social stress via γδ T cells.^108^ Dectin-1 mediates the signaling of colonic interleukin-17-producing γδ T cells (γδ17 T cells) and their accumulation in the meninges, which results in stress-susceptible behavior.^108^

Major depressive disorder is also considered a microglial disease. Structural and functional impairment of microglia caused by intense inflammatory activation or cellular senescence can lead to depression and deficits associated with neural plasticity and neurogenesis.^109^ Furthermore, the microglia/Dectin-1 association is present in psychiatric disorders such as depression.^109^ Most studies evaluating the antidepressant potential of Dectin-1 involve microglial populations and anti-inflammatory effects. In depression, inhibition of the Dectin-1 receptor prevents microglial activation and astrocyte proliferation, reducing the expression of IL-1β and TNF-α and increasing IL-10 and BDNF.^10^ However, studies indicate that chronic inflammation induction models increase the levels of inflammatory cytokines in microglia mediated by Dectin-1 receptors.^110^ This suggests a dual role for Dectin-1 that still needs to be elucidated in psychiatric disorders.

### 4.4 Neurodegenerative Diseases

Neurodegenerative diseases disrupt motor and cognitive functions, impacting patient’s quality of life. Current pharmacological therapies for these conditions aim to alleviate symptoms as the disease progresses; however, their effectiveness is limited.^111^ Widely documented evidence highlights the Dectin-1 receptor as playing a key role in the development of these neuropathologies, particularly in Parkinson’s disease (PD) and Alzheimer’s disease (AD).^12,112^ The upregulation of the CLEC7A gene is involved in the development and progression of neurodegenerative diseases such as AD and PD.^112^ According to Zhao et al. (2023), the Dectin-1 receptor is involved in neuroinflammation associated with the development of AD.^12^ Microglial Dectin-1 mediates inflammatory responses to beta-amyloid (Aβ) protein.^12^ Aβ42 binds to Dectin-1, leading to its homodimerization and activation of the Syk/NF-kB, pathway to induce inflammatory factors.^12^ Aligned with this, elevated levels of Clec7a and p-Syk in the ventral hippocampus of tauopathy mice further support the involvement of the Clec7a–Syk signaling pathway in this disease model.^113^ Clec7a has also been associated with activation and synaptic loss in microglia cells. Notably, inhibition of Clec7a with Laminarin has been shown to ameliorate memory deficits in tauopathy mice.^113^ Thus, CLEC7A is already used as a reference microglial marker gene associated with AD.^114^ These data suggest the critical role of microglial Dectin-1 as a new direct receptor for Aβ42, offering therapeutic strategies for neuroinflammation in AD.^114^ Supporting this, studies have shown that negative modulation of CLEC7A improves microglial activation in AD.^115^ Thus, CLEC7A emerges as a therapeutic target to regulate microglial activation in AD.

In PD, there is high expression of the CLEC7A gene in the substantia nigra and striatum of PD model rats, mainly localized in microglia.^112^ According to Chen et al. (2023), CLEC7A knockdown restricted neuroinflammation by suppressing the release of inflammatory factors such as IFN-γ, TNF-α, IL-1β, IL-18, and IL-6, resulting in increased expression of arginase-1 (M2 polarization) and decreased expression of iNOS, (M1 polarization).^112^ These results were also observed in the LPS-induced inflammatory PD rat model.^112^ Furthermore, in vitro evidence from the same study demonstrated that α-synuclein fibrils induced upregulation of CLEC7A expression and microglial polarization to a pro-inflammatory state in BV2 cells, leading to increased cytokine release.^112^ CLEC7A knockdown reversed these changes and induced a shift to an anti-inflammatory phenotype in BV2 microglial cells.^112^ Similarly, in an LPS-induced PD model, Xue et al. (2025) observed upregulation of Dectin-1 in the neuroinflammation of the substantia nigra in mice and BV2 microglia cells, which was associated with TLR4 induction. Dectin-1 upregulation was also related to microglial activation.^13^ In this study, inhibition of Dectin-1 by laminarin attenuated LPS-induced motor impairments and dopaminergic neuronal loss in mice.^13^ Furthermore, Dectin-1 inhibition promoted a shift in microglial phenotype from M1 to M2 in the substantia nigra of mice and BV2 cells, which is suggested to occur through the Syk/NF-kB signaling pathway, as laminarin treatment downregulated p-P65/P65 and p-Syk/Syk levels.^13^ In BV2 cells, the relationship between Dectin-1 and M1 microglial activation, as well as the production of inflammatory mediators, was demonstrated through knockdown of this receptor, which inhibited the upregulation of COX-2 and iNOS induced by LPS.^13^ The authors also showed that TLR4/NF-κB signaling regulates Dectin-1 expression on M1 microglia: BV2 cells pretreated with TLR4 or NF-κB inhibitors before LPS administration showed decreased Dectin-1 expression.^13^ The role of Dectin-1 in neuroinflammation was also demonstrated through administration of its agonist, d-Zymosan, into the substantia nigra of mice, which induced dopaminergic neuron degeneration and behavioral impairments. In this context, Dectin-1 also upregulated TLR4 expression in microglia.^13^ Taken together, the results of Xue et al. (2025) demonstrate the important influence of the Dectin-1 receptor in microglia-mediated neuroinflammation in PD.^13^ Moreover, recent evidence highlights the selective enrichment of the CLEC7A gene promoted by Dark Microglia (DM).^116^ The preclinical PD model revealed a higher number of CLEC7A-positive cells, particularly in the DM population.^116^ Most of these cells acquired an amoeboid phenotype and clustered in affected animals.^116^ Therefore, these results suggest that CLEC7A mediates signaling processes that mitigate neuroinflammation in PD.^116^

Thus, activation of the Dectin-1 receptor has dual effects, being pro-inflammatory or anti-inflammatory depending on the cell specificity, nature of the ligands involved, activation dynamics and pathological context in which this signaling is inserted. In psychiatric disorders, such as depression, ligands such as β-glucans appear to play a crucial role in the anti-inflammatory response, while in neurodegenerative diseases, the main ligands Aβ and α-synuclein may elicit different signaling pathways after receptor activation particularly in microglial cells, which are key mediators of the neuroimmune response in the CNS.^12,83^ Furthermore, in depression, the effect of Dectin-1 is mainly interconnected with microglia as well as astrocytes and γδ T cells, while in AD and PD, the exacerbated activation of Dectin-1 appears to be related to the presence of aggregated proteins such as Aβ and α-synuclein.^12,108,112^ Furthermore, temporal dynamics also appear to influence the effect of Dectin-1. While in depression, Dectin-1 activation appears to be moderate and controlled, promoting immunomodulatory and neuroprotective effects, in neurodegeneration, chronic activation favors a chronic inflammatory profile.^12,105^ Although the exact mechanisms still need to be elucidated, evidence suggests that the anti-inflammatory effect of Dectin-1 in depression is due to the modulation of the Syk/NF-kB pathway and by inhibiting the activation of TLR4.^83,105^ Thus, there is an increase in the anti-inflammatory response from the expression of IL-10 and BDNF to promote neural plasticity.^10,109^ While in AD and PD, the activation of Dectin-1 by protein aggregates appears to favor the induction of pro-inflammatory profiles of microglia, which can contribute with the progression of neurodegenerative disorders.^112^ Therefore, despite the duality of the Dectin-1 signaling effects according to the pathological scenarios, more studies are still needed to fully elucidate the dynamics of these receptor in health and CNS disease (Figure 4).

## 5 Challenges and Futures Perspectives

Despite the growing interest in Dectin-1 as a promising therapeutic target for CNS disorders, pharmacological challenges remain to be addressed. A primary obstacle is the restricted permeability of the blood-brain barrier (BBB) that serves as a protective structure to maintain brain homeostasis.^117,118^ Molecular size and lipophilicity determine the ability of a drug to penetrate into the brain, as well as its active persistence at safe concentrations upon crossing.^117^ Thus, conventional systemic delivery may have inefficient bioavailability in the brain and, in addition, increase the risk of peripheral effects due to the expression of Dectin-1 in immune cells outside the CNS.^29,118^ Promising drug delivery advances are emerging to overcome these obstacles, such as the use of targeting vectors, structural modification of drugs to increase lipophilicity, receptor-specific monoclonal antibodies, as well as the use of nanoparticle carriers.^118–120^ Research points to the use of nanoparticles, such as zein-polydopamine or lipid-based nanoparticles, to increase BBB permeability and optimize cellular uptake of microglia, one of the cells that express Dectin-1.^121^ Alternative pathways have also gained strength to bypass the BBB and reach specific neuroimmune pathways.^81^ However, the particularity of these alternatives and the long-term effects for receptor modulation in complex disorders still need to be elucidated. Furthermore, additional studies in robust models are needed to determine the effects of receptor modulation with agonist and antagonist molecules in order to optimize administration platforms and validate the therapeutic potential of Dectin-1 in vivo and advance to clinical studies.

**Figure 4:**
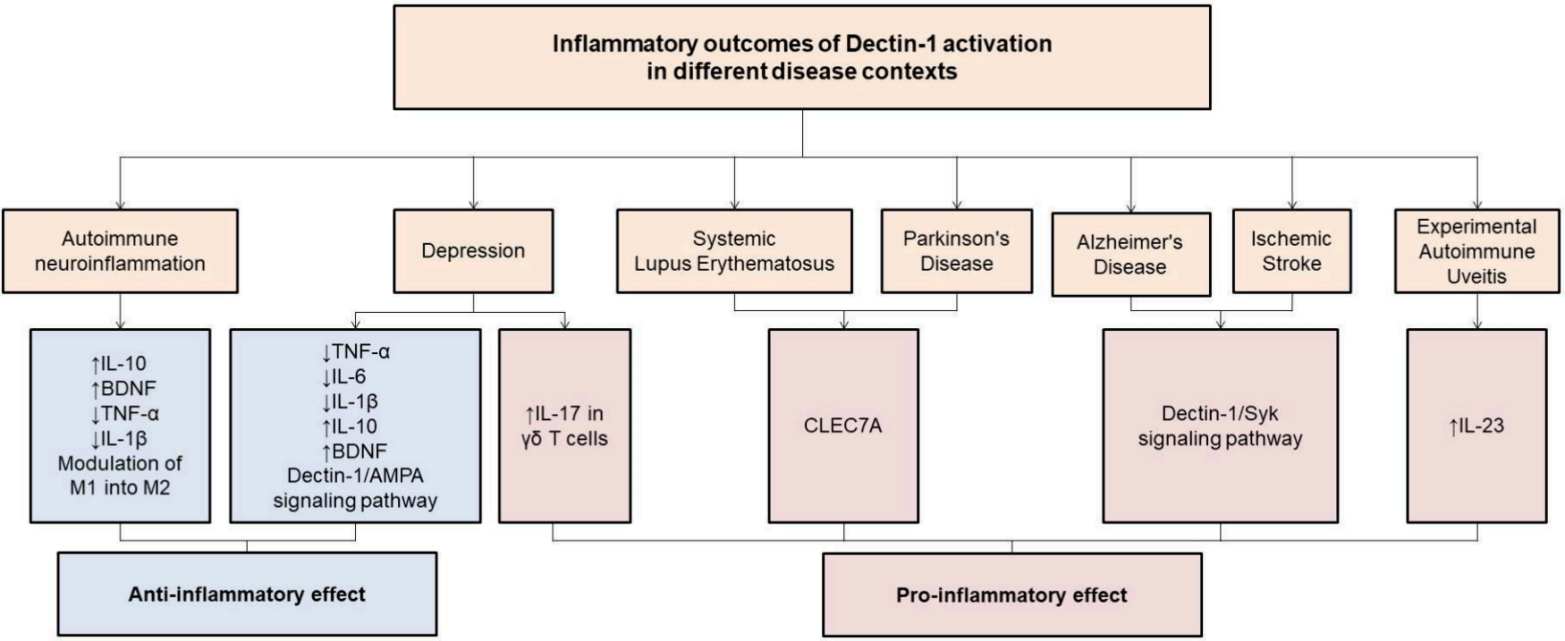
This diagram illustrates the dual role of Dectin-1 activation in various disease models, highlighting both anti-inflammatory and pro-inflammatory responses. In autoimmune neuroinflammation and depression, Dectin-1 activation is associated with anti-inflammatory outcomes, including increased IL-10 and BDNF levels, reduced pro-inflammatory cytokines, modulation of microglial/macrophage polarization from the pro-inflammatory M1 to the anti-inflammatory M2 phenotype, and involvement of the AMPA signaling pathway. In contrast, in systemic lupus erythematosus, Parkinson’s disease, Alzheimer’s disease, ischemic stroke, and experimental autoimmune uveitis, Dectin-1 or its gene (CLEC7A) is implicated in pro-inflammatory effects, such as enhanced IL-17 production by γδ T cells, IL-23 elevation, and activation of the Syk signaling cascade.

## 6 Conclusions

This review explored the multifaceted role of Dectin-1 in various neuropsychiatric disorders and its association with DAMs. Evidence suggests that Dectin-1 activation can either protect or damage the brain. Understanding the mechanisms of action of Dectin-1, its modulation pathways, and its effects on microglial cells is essential for the discovery of new therapeutic agents. Future research should delve deeper into pathological contexts where Dectin-1 may help mitigate or slow disease progression.

## Abbreviations

Aβ42: amyloid beta peptide 1–42
AD: Alzheimer’s disease
AKT: protein kinase B
AMPA: α-amino-3-hydroxy-5-methyl-4-isoxazolepropionic acid
APOE: apolipoprotein E
ARG-1: arginase-1
BBB: blood-brain barrier
BCL10: B-cell lymphoma 10
BDNF: brain-derived neurotrophic factor
CA^2+^: Calcium ions
CARD9: caspase recruitment domain-containing protein 9
CAT: catalase
Clec2: C-type Lectin-like Receptor 2
Clec7a: gene that encodes Dectin-1 receptor
CLR: C-type lectin receptors
CNS: central nervous system
CRD: carbohydrate recognition domain
CSDS: chronic social-defeat stress test
DAM: disease-associated microglia
DAMPs: damage-associated molecular patterns
DM: dark micróglia
EAE: experimental autoimmune encephalomyelitis
EAU: experimental autoimmune uveitis
GLP: *Ganoderma lucidum* polysaccharide
GSH-Px: glutathione peroxidase
hemITAM: immunoreceptor tyrosine-based inhibitory motif
HIV: human immunodeficiency virus
HO: heme oxygenase
IFN: interferon
IL: interleukin
IKKb: inhibitory kinase beta
iNOS: inducible nitric oxide synthase
IRF-5: interferon regulatory factor 5
ITAM: immunoreceptor tyrosine-based activation motif
LDH: lactate dehydrogenase
LNT: lentinan
LPS: Lipopolysaccharide
MALT1: mucosa-associated lymphoid tissue 1
MDP: muramyl dipeptide
moDCs: monocyte-derived dentritie cells
mRNA: mRNA
NFAT: nuclear Factor of Activated T-cells
NF-kB: nuclear factor kappa B
NLRP3: NOD-like receptor family, pyrin domain containing 3
Nrf2: nuclear factor erythroid 2-related factor 2
OGD/R: oxygen-glucose deprivation/reperfusion
Osm: Oncostatin M cytokine
p65: subunit of the nuclear factor kappa B
PAMPs: pathogen associated molecular patterns
PIC: piceatannol
PLCγ2: hospholipase C gamma 2
PD: Parkinson’s disease
PI3Ks: phosphatidylinositol 3-kinase
PRR: pattern recognition receptors
ROSs: reactive oxygen specie
SLE: systemic lupus erythematosus
SN: substantia nigra
SOD: superoxide dismutase
SRC: protein tyrosine kinase family
Syk: tyrosine-protein kinase
TAK1: transforming Growth Factor b-Activated Kinase 1
TLRs: toll-like receptors
TNF: tumor necrosis factor
TREM-2: triggering receptor expressed on myeloid cells 2
WGP: whole glucan particles
WT: Wild-Type
